# *Bacillus mojavensis* dxk33 Modulates Rhizosphere Microbiome and Suppresses Root Rot in *Cunninghamia lanceolata*

**DOI:** 10.3390/microorganisms14010034

**Published:** 2025-12-22

**Authors:** Xiaokang Dai, Pengfei Yang, Chuan Zhou, Zebang Chen, Shuying Li, Tianhui Zhu

**Affiliations:** 1 College of Forestry, Sichuan Agricultural University, Chengdu 611130, China; 2 Forest Ecology and Conservation in the Upper Reaches of the Yangtze River Key Laboratory of Sichuan Province, Chengdu 611130, China; 3 Sichuan Mt. Emei Forest Ecosystem National Observation and Research Station, Chengdu 611130, China; 4 College of Engineering, University of Georgia, Chapel, Herty Dr, Athens, GA 30602, USA

**Keywords:** soil-borne disease, plant growth-promoting rhizobacteria, microbial community engineering, soil microbial dysbiosis, biological control, fusarium solani, functional prediction

## Abstract

Soil-borne pathogens cause devastating root rot diseases in forest ecosystems, often by inducing dysbiosis in the rhizosphere microbiome. While antagonistic bacteria can suppress disease, their effects frequently extend beyond direct inhibition to include ecological restructuring of resident microbial communities. However, the causal relationships between such microbiome restructuring and disease suppression in tree species remain poorly understood. Here, we show that the antagonistic bacterium *B. mojavensis* dxk33 effectively suppresses *F. solani*-induced root rot in *C. lanceolata*, and that this disease suppression coincides with a partial reversal of pathogen-associated dysbiosis in the rhizosphere. Inoculation with dxk33 significantly promoted plant growth and reduced the disease index by 72.19%, while concurrently enhancing soil nutrient availability and key C-, N- and P-cycling enzyme activities. High-throughput sequencing revealed that dxk33 inoculation substantially reshaped the rhizosphere microbiome, counteracting the pathogen’s negative impact on microbial diversity and coinciding with a shift toward a more stable community structure. Under pathogen stress, dxk33 enriched beneficial bacterial taxa such as *Pseudomonas* and *Sphingomonas* and suppressed pathogenic fungi while promoting beneficial fungi such as *Mortierella*. Linear discriminant analysis and functional prediction further indicated that dxk33 remodeled ecological guilds enriched for mycorrhizal and saprotrophic fungi, and reactivated bacterial metabolic pathways and signaling networks that were suppressed by the pathogen. Taken together, our findings are consistent with a multi-tiered mode of action in which direct antagonism by *B. mojavensis* dxk33 operates alongside associated changes in the rhizosphere microbiome that resemble a disease-suppressive state, although the present experimental design does not allow a strictly causal role for microbiome reconfiguration in disease suppression to be established. This study provides a mechanistic framework for understanding how microbiome engineering may mitigate soil-borne diseases in perennial trees and highlights the potential of targeted microbial interventions for sustainable forest management.

## 1. Introduction

Rhizosphere, the narrow zone of soil directly influenced by plant roots, represents one of the most dynamic and critical interfaces on Earth, governing nutrient cycling and carbon dynamics characterized by high C turnover and priming effects that may increase soil C mineralization rather than net C sequestration, ultimately shaping overall ecosystem productivity [1,2]. Far from being a passive substrate, the rhizosphere is colonized by a diverse consortium of microorganisms, including bacteria, fungi, archaea and protists, that form complex, interdependent between them, and associations with their host plant. This community, collectively termed the rhizosphere microbiome, consists of microorganisms inhabiting the narrow soil zone surrounding plant roots. Recently, the rhizosphere microbiome has been conceptualized as an “extended phenotype” of the host plant in the sense that plant genetic variation in traits such as root exudation, immune responses and root architecture can influence the composition and functioning of associated microbial communities. However, microbiome assembly is not determined by host genetics alone; strong environmental filtering by soil physicochemical properties, climate, management practices and the local microbial species pool also constrains which microorganisms can be recruited. As a result, host genetic control over the rhizosphere microbiome is expressed only within the limits set by these abiotic and biotic conditions [3]. The intricate dialogue between plants and microbes facilitates a range of essential ecological services, ranging from enhancing nutrient acquisition and soil structure to activating plant defense responses such as induced systemic resistance (ISR) and systemic acquired resistance (SAR) against soil-borne pathogens. Consequently, the composition and functional integrity of the rhizosphere microbiome are fundamental determinants of plant fitness, and by extension, the sustainability of terrestrial ecosystems. [4,5]. A stable and diverse microbiome acts as a first line of defense against soil-borne diseases by competing with pathogens for resources and niche space and producing a suite of antimicrobial compounds that suppress invasion [6,7]. Understanding the mechanisms that maintain this beneficial microbiome is therefore paramount for managing plant health in both natural and managed ecosystems [8].

However, this beneficial plant–microbe association is fragile and can be critically disrupted by external drivers such as intensive monoculture, soil degradation and climatic stress, which may shift a dysbiotic soil microbiota into a disbiotic state and alter the ecological guild structure of facultative organisms such as *Fusarium* spp. that can behave as endophytes in healthy plants. These pathogens act not merely as direct infectious agents but as powerful community modulators that trigger a state of dysbiosis, i.e., a fundamental shift in the rhizosphere microbiome from a healthy, resilient state to one that is destabilized and conducive to disease [9]. Beyond directly infecting root tissues and causing rot, necrosis and wilting, soil-borne pathogen invasion can also act as a strong ecosystem filter that reconfigures the resident microbial community. It often suppresses beneficial taxa and favors the proliferation of opportunistic and pathogenic microorganisms, thereby shifting the rhizosphere from a stable, health-associated configuration towards a dysbiotic state. In the context of this study, we therefore adopt an operational, quantitative definition of rhizosphere dysbiosis in *C. lanceolata*. The uninoculated control (CK) is used as the baseline “healthy” state, characterized by relatively high fungal and bacterial Shannon diversity, low among-replicate dispersion in β-diversity, and a functional guild structure dominated by saprotrophic and mycorrhizal fungi in the absence of disease symptoms. By contrast, we classify the rhizosphere as “dysbiotic” when pathogen challenge leads to: (i) significant (*p* < 0.05) reductions in fungal and/or bacterial Shannon diversity; (ii) pronounced shifts in β-diversity relative to the healthy baseline, reflected by increased Bray–Curtis dissimilarities and clear separation in ordination space; and (iii) a reconfiguration of functional guilds from saprotrophic/mycorrhizal dominance toward pathogenic and parasitic taxa, accompanied by an increased disease index. These quantitative criteria provide baseline metrics for distinguishing pathogen-induced community disruption from normal background variation and for consistently comparing “healthy” versus “dysbiotic” rhizosphere states in *C. lanceolata* [10,11]. Such pathogen-induced dysbiosis can compromise essential rhizosphere functions (e.g., nutrient cycling and microbiome-mediated suppression of pathogens), thereby potentially exacerbating disease progression and weakening the host plant. [12,13]. The resulting breakdown of the rhizosphere’s natural suppressive capacity paves the way for the establishment of severe disease epidemics, posing a major threat to plant health and ecosystem productivity.

Considering the detrimental impact of pathogen-induced dysbiosis, there is a growing paradigm shift towards managing soil-borne diseases through targeted microbiome engineering to steer microbial communities toward a stable, beneficial and suppressive state [14]. Within this framework, the application of antagonistic bacteria is not viewed merely as a direct biocontrol tactic but as a strategic ecological intervention to reboot the functional integrity of the rhizosphere. Certain beneficial microorganisms, particularly strains of the genus *Bacillus* [15,16], are emerging as powerful catalysts for this process due to their metabolic versatility, ability to produce a diverse arsenal of antimicrobial compounds and their capacity to act as keystone taxa that exert a disproportionate influence on microbiome structure and function [16]. By competing for resources, producing antibiotics and potentially modulating host defense pathways, these introduced antagonists can selectively suppress pathogens while simultaneously enriching for a broader consortium of beneficial indigenous microbes [17]. This indirect, community-wide effect is increasingly recognized as a fundamental mechanism behind successful biological control, transforming a dysbiotic environment into one that is resilient to pathogen invasion [18]. The ultimate goal of such an approach is not just to eliminate a single pathogen, but to foster a self-sustaining, disease-suppressive soil microbiome, thereby providing durable plant protection.

Microbiome engineering, understood as the targeted manipulation of host-associated microbial communities to enhance plant performance, has been proposed as a promising strategy to manage soil-borne diseases in intensively managed systems [19]. However, several key challenges still limit the translation of this concept into practical applications. First, the long-term persistence and temporal dynamics of engineered rhizosphere communities remain uncertain, especially in long-lived woody species such as *C. lanceolata* (Lamb.) Hook. (Pinopsida: Cupressaceae), where seasonal dynamics, stand development and management interventions can gradually erode or reshape inoculant effects [20]. Second, the dose–response relationships between bacterial inoculant abundance and disease suppression or growth promotion are still poorly constrained; both under- and over-application may lead to suboptimal performance, unintended shifts in native microbiota or reduced cost-effectiveness [21]. Third, the efficacy of biocontrol agents is strongly context-dependent, because soil type, nutrient status, pH, moisture regime and background microbial diversity can all modify the establishment and function of introduced strains. These considerations highlight the need for carefully designed experiments that not only demonstrate proof-of-concept under controlled conditions, but also begin to define the temporal persistence, dose–response behavior and environmental context-dependence of microbiome-based interventions in *C. lanceolata* plantations. To translate the principles of microbiome engineering into tangible solutions for forest ecosystems, it is essential to study these interactions within ecologically and economically important tree species. *C. lanceolata*, a cornerstone of afforestation and timber production in China, represents such a system [22]. This fast-growing conifer plays a major role in maintaining forest structure and contributing to carbon dynamics, yet its cultivation is severely threatened by soil-borne diseases, with root rot involving members of the *F. solani* (Mart.) Sacc. (*Sordariomycetes*: *Nectriaceae*) species complex being a major constraint on plantation health and productivity. *F. solani*, a member of a species complex with context-dependent lifestyles that can range from endophytic to pathogenic, is frequently associated with root rot and with shifts in root- and soil-associated fungal communities in susceptible hosts, although its precise causal role in microbiota disruption remains to be fully resolved [23]. The susceptibility of *C. lanceolata* to these fungi underscores a significant vulnerability in its management. Notably, tree-to-tree differences in physiological performance and disease tolerance are commonly observed in *C. lanceolata* stands, a pattern that may be driven by underlying variation in their associated rhizosphere microbiomes [24]. While previous studies have confirmed the severe impact of *F. solani* on *C. lanceolata* health [25], the potential for beneficial bacteria to counteract this threat by inducing and steering a protective microbiome remains a critical, unexplored frontier. Therefore, the *C. lanceolata–F. solani* pathosystem provides a highly relevant and compelling model to investigate the mechanisms through which an antagonistic bacterium can remediate dysbiosis and restore rhizosphere functionality in a perennial forest tree. In this study, we used the antagonistic bacterium *B. mojavensis* dxk33 as a model biocontrol strain. This Gram-positive, spore-forming rhizobacterium was originally isolated from the rhizosphere of healthy *C. lanceolata* seedlings during a culture-dependent screening of microorganisms associated with healthy and diseased plantations. In vitro dual-culture assays and liquid co-cultures showed that dxk33 exhibits strong antagonistic activity against *F. solani* and several other soil-borne fungal pathogens. In earlier work, dxk33 showed strong antagonistic activity against *Fusarium solani* in dual-culture assays, suggesting a direct biocontrol potential. Consistent with this, greenhouse pot bioassays conducted in the present study (six biological replicates per treatment) provided quantitative evidence that a preventive application of dxk33 mitigated *F. solani*–induced root rot and improved seedling performance. Specifically, the disease index was reduced from 79.38 ± 0.83 in the pathogen-only treatment (Fs) to 24.66 ± 1.12 in the dxk33 + Fs treatment, corresponding to a 68.93% control efficacy (Table 1). Moreover, dxk33 alone promoted seedling growth relative to the uninoculated control, as reflected by increased plant height (42.49 ± 1.14 cm vs. 33.47 ± 0.82 cm) and higher biomass (Table 1). Taken together, these results support the view that dxk33 combines robust suppression of a soil-borne fungal pathogen with plant growth–promoting effects, providing a clear rationale for selecting *Bacillus mojavensis* dxk33 as the focal strain for microbiome engineering in this study.

Despite the recognized importance of the rhizosphere microbiome in plant health, critical knowledge gaps persist regarding the mechanistic basis through which an introduced antagonistic bacterium can remediate pathogen-induced dysbiosis and restore ecosystem function in tree species [26]. Specifically, there is a lack of causal understanding of how a specific beneficial bacterium such as *B. mojavensis* restructures the entire rhizosphere community in the face of a pathogen challenge [11]. Furthermore, it remains unclear how these structural shifts translate into functional changes in the metabolic potential and ecological guilds of microbiomes, and how these changes are integrated with alterations in soil biochemical properties to collectively suppress disease [10]. Bridging these gaps requires an integrated approach that couples plant phenotyping with comprehensive profiling of the soil nutrient environment, microbial taxonomy and predicted function. Therefore, we hypothesized that inoculation with the antagonistic bacterium *B. mojavensis* dxk33 would suppress root rot and would be associated with a partial reversal of the dysbiosis induced by *F. solani*, thereby restructuring the microbial community toward a more functionally resilient and suppressive state and enhancing soil nutrient cycling. To test this hypothesis, we designed a controlled study with the following objectives.

To systematically test our central hypothesis, this study was designed to pursue the following interconnected objectives: First, to quantify the direct biocontrol efficacy of *B. mojavensis* dxk33 against *F. solani*-induced root rot and its concomitant growth-promoting effects on *C. lanceolata* seedlings. Second, to assess how dxk33 inoculation, both in the presence and absence of the *F. solani*, influences rhizosphere soil nutrient status and the activities of key enzymes integral to carbon, nitrogen, and phosphorus cycling. Third, to characterize the structural and compositional changes in both the fungal and bacterial components of the rhizosphere community induced by these treatments using high-throughput sequencing of the ITS1 and 16S rRNA gene regions. Fourth, to identify the specific microbial taxa and model functional guilds that are differentially enriched by dxk33 inoculation under pathogen stress, thereby pinpointing the key players associated with the suppressive state. Finally, to predict the functional consequences of this community restructuring by inferring the metabolic potential of the bacterial community and the ecological roles of the fungal community, thereby linking taxonomic shifts to ecosystem functionality. Through this multi-faceted approach, we aim to propose a mechanistic model to understand how a targeted bacterial inoculant may engineer a disease-suppressive rhizosphere microbiome in a key forest tree species.

## 2. Materials and Methods

### 2.1. Experimental Materials

To minimize genetic background variation, three-year-old seedlings of *C. lanceolata* used in this study were collected from a uniform genetic source at the Hongya Forest Farm in Sichuan Province, China (102°49′–103°32′ E, 29°24′–30°00′ N). The specific biocontrol strain used in this experiment, *B. mojavensis* dxk33, was previously isolated from the rhizosphere soil of healthy *C. lanceolata* seedlings during a culture-dependent screening of rhizosphere microorganisms, where it was identified as a promising antagonist of soil-borne pathogens. In earlier work, dxk33 showed strong inhibitory activity against *F. solani* in dual-culture assays. Consistent with this antagonistic potential, greenhouse pot bioassays conducted in the present study (six biological replicates per treatment) further demonstrated that a preventive soil-drench application of dxk33 markedly mitigated *F. solani*–induced root rot and improved seedling performance. Specifically, the disease index decreased from 79.38 ± 0.83 in the pathogen-only treatment (Fs) to 24.66 ± 1.12 in the dxk33 + Fs treatment, corresponding to a 68.93% control efficacy (Table 1). Moreover, dxk33 alone promoted seedling growth relative to the uninoculated control, as indicated by increased plant height (42.49 ± 1.14 cm vs. 33.47 ± 0.82 cm) and higher fresh and dry biomass (Table 1). Collectively, these results support the selection of *B. mojavensis* dxk33 as the focal strain for microbiome engineering in this study because it combines disease-suppressive activity against a soil-borne fungal pathogen with measurable plant growth–promoting effects. In contrast, the pathogenic *F. solani* strain used here was isolated from diseased root tissues of *C. lanceolata* seedlings exhibiting typical root rot symptoms. Pathogenicity of the *F. solani* isolate toward *C. lanceolata* was verified following Koch’s postulates. Briefly, the fungus was purified by single-spore isolation on PDA, and a conidial suspension was prepared and adjusted to 1 × 10^7^ spores mL^−1^. Ten healthy three-year-old *C. lanceolata* seedlings were inoculated by soil drench, while ten mock-inoculated seedlings received sterile water containing 0.05% (*v*/*v*) Tween 20 and served as controls. Inoculated seedlings subsequently developed characteristic root rot symptoms, which became apparent at 30 days post inoculation, whereas no typical symptoms were observed in the mock-inoculated controls. The pathogen was then re-isolated from symptomatic root tissues by plating surface-sterilized root segments on PDA. Re-isolated colonies were identified based on colony and microscopic morphology, and their identity was further confirmed by ITS sequencing: the ITS region was amplified using primers ITS1/ITS4, Sanger sequenced, and the resulting sequences showed ≥99% identity to *F. solani* reference sequences in GenBank.

### 2.2. Plant Materials, Experimental Design, and Treatments

**Figure 1:**
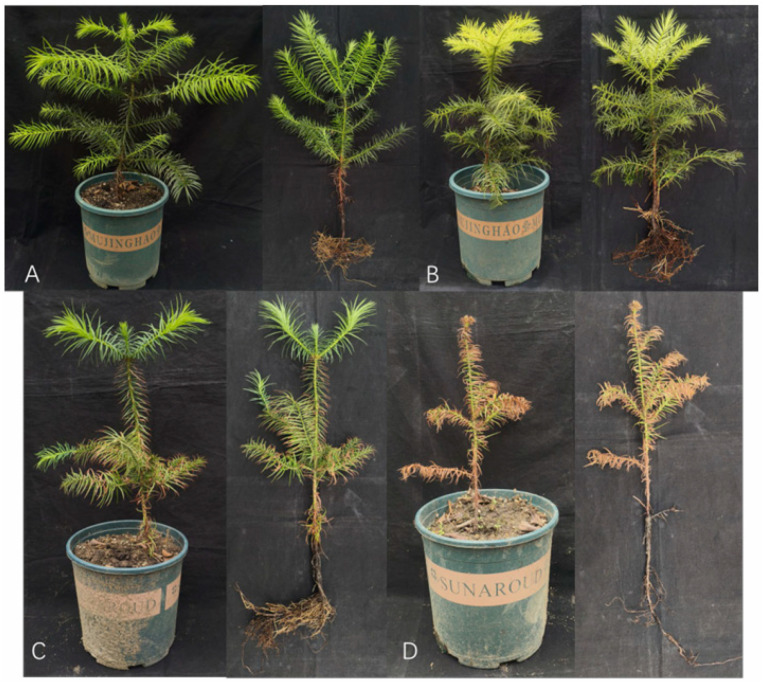
Effect of *Bacillus mojavensis* dxk33 on *Cunninghamia lanceolata* root rot in pot experiments. (**A**) Seedlings treated with *B. mojavensis* dxk33 alone showing vigorous growth and healthy root systems. (**B**) Untreated control (CK) seedlings exhibiting normal growth. (**C**) Seedlings co-treated with *B. mojavensis* dxk33 and *Fusarium solani* (dxk33 + Fs) showing significantly reduced disease symptoms compared to pathogen-only treatment. (**D**) Seedlings treated with *F. solani* alone (Fs) showing severe root rot symptoms including chlorosis, defoliation, and root necrosis.

**Figure 2:**
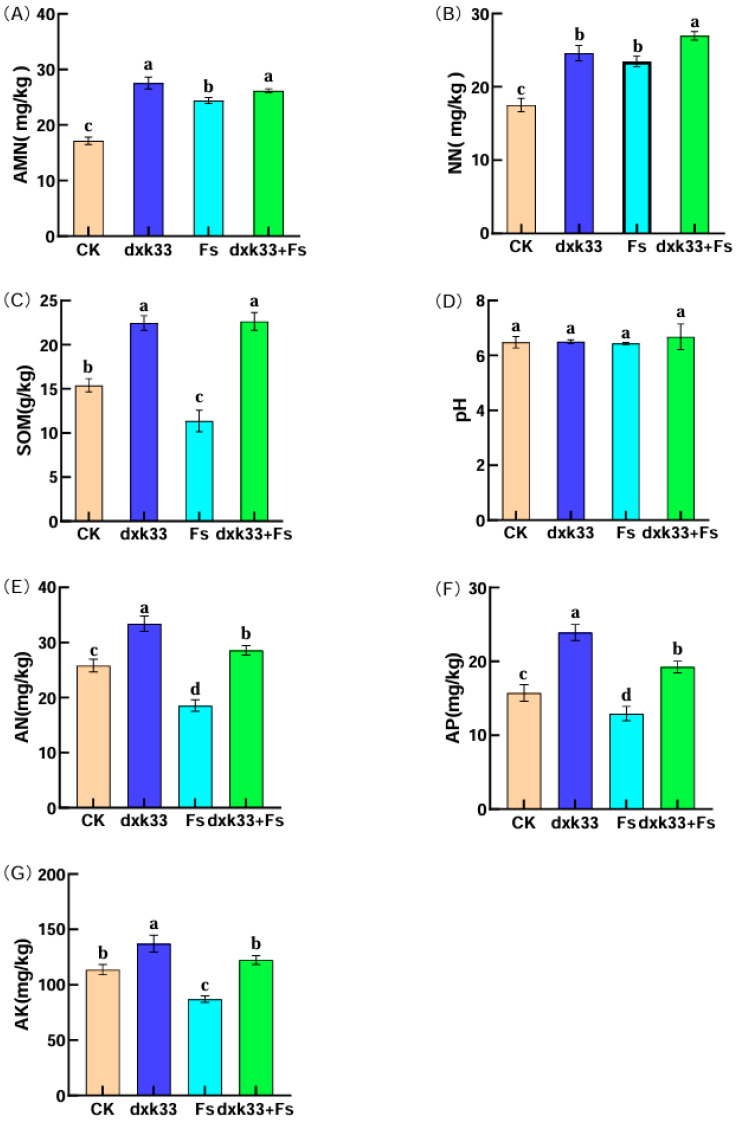
Effects of *B. mojavensis* dxk33 and *Fusarium solani* (Fs) inoculation on soil nutrient status in the rhizosphere of *C. lanceolata*. Panels (**A**–**G**) show changes in ammonium nitrogen (AMN), nitrate nitrogen (NN), soil organic matter (SOM), soil pH, available nitrogen (AN), available phosphorus (AP), and available potassium (AK) contents under different treatments. Different letters indicate significant differences among treatments (*p* < 0.05, generalized linear models followed by Wald pairwise tests). Each treatment included six replicates, and values are presented as means ± standard error. CK: control; dxk33: inoculated with *B. mojavensis* dxk33; Fs: inoculated with *F. solani*; dxk33 + Fs: co-inoculated with *B. mojavensis* dxk33 and *F. solani*.

**Figure 3:**
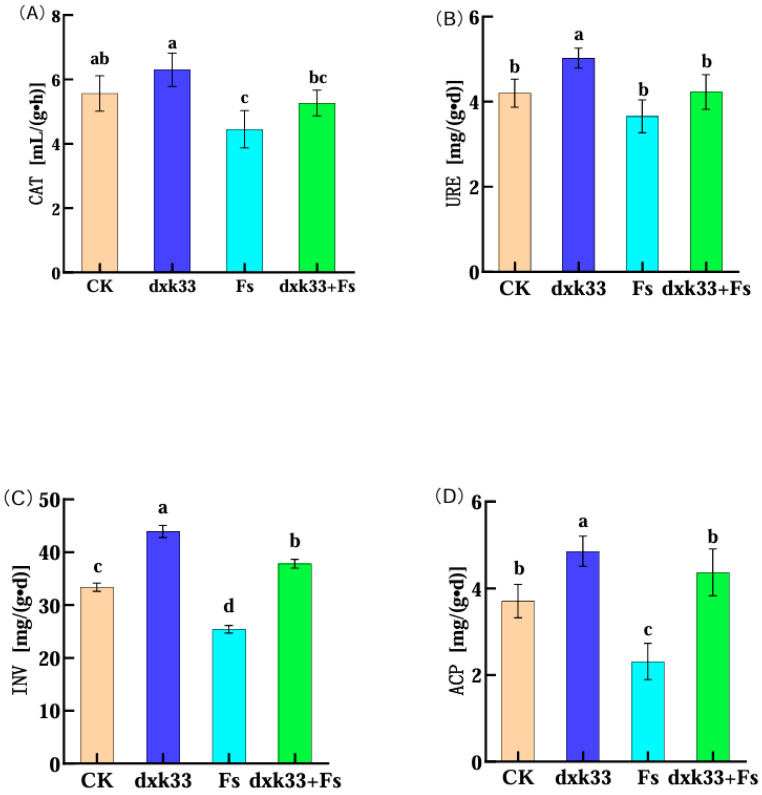
Effects of *Bacillus mojavensis* dxk33 and *Fusarium solani* (Fs) inoculation on rhizosphere soil enzyme activities in *Cunninghamia lanceolata*. Panels (**A**–**D**) show the activities of catalase (CAT), urease (URE), invertase (INV) and acid phosphatase (ACP), respectively, in rhizosphere soil under the different treatments. Different letters above the bars denote significant differences among treatments (one-way ANOVA followed by Tukey’s HSD test, *p* < 0.05). Each treatment comprised six biological replicates, and data are presented as mean ± standard error. CK, uninoculated control; dxk33, inoculated with *B. mojavensis* dxk33; Fs, inoculated with *F. solani*; dxk33 + Fs, co-inoculated with *B. mojavensis* dxk33 and *F. solani*.

In this greenhouse experiment, three-year-old *Cunninghamia lanceolata* seedlings were transplanted singly into plastic pots, each filled with 7 kg of air-dried bulk soil collected from uncultivated land in the Bamianshan Forest Farm area (Meishan, China). The regional zonal soil in this area is typically classified as yellow soil (often referred to locally as mountain yellow soil), and the bulk soil used here was of this yellow-soil type. The soil was gently crumbled, passed through a 2 mm sieve to remove stones and plant residues, and used as non-sterile bulk soil without sterilization or pasteurization to preserve its indigenous physicochemical properties and microbial community. The soil was gently crumbled, passed through a 2 mm sieve to remove stones and plant residues, and used as non-sterile bulk soil without sterilization or pasteurization in order to maintain its indigenous physicochemical properties and microbial community. To minimize background microbiome variation among pots at the start of the experiment, soil collected from the site was combined, thoroughly homogenized as a single composite batch, and then evenly allocated to all pots. Pots were subsequently randomly assigned to treatments, and their positions were randomized during the experiment to minimize positional effects. Each treatment (CK, dxk33, Fs and dxk33 + Fs) comprised six biological replicates, with one seedling per pot treated as an independent experimental unit. This sample size was chosen a priori, informed by previous greenhouse studies of soil-borne diseases in forest trees and other crops that used comparable numbers of plants per treatment, as well as preliminary trials in the *C. lanceolata–F. solani* pathosystem in which differences in disease index and biomass were consistently detectable at this level of replication. The selected number of replicates reflects a balance between statistical precision and practical constraints associated with greenhouse space and the labor-intensive processing of soil physicochemical, enzymatic and microbiome samples. The greenhouse experiment (six biological replicates per treatment, one seedling per pot) was designed a priori to balance statistical resolution with the practical constraints of a multi-endpoint mechanistic study (plant traits, soil chemistry/enzymes, and microbiome profiling). Importantly, the main outcomes showed large effect sizes relative to within-treatment variability (e.g., disease index and key growth traits; Table 1; Figure 1, Figure 2 and Figure 3), and the corresponding between-treatment contrasts were consistently detectable at this replication level, which is also in line with comparable greenhouse studies of soil-borne diseases. After transplanting, seedlings were maintained for 90 days in a greenhouse at Sichuan Agricultural University for acclimation and were then transferred to a controlled-environment growth chamber maintained at 24 °C and 65% relative humidity with a 14 h/10 h light–dark photoperiod for a 30-day inoculation experiment. A constant temperature regime was chosen to minimize environmental variability among treatments and to approximate the mean growing-season temperature in the study region, thereby allowing us to focus on the effects of pathogen and bacterial inoculation on plant performance and rhizosphere microbiome responses. For preparation of the bacterial inoculum, *B. mojavensis* dxk33 was grown in LB broth at 28 °C with shaking at 180 rpm for 18–20 h until late exponential/early stationary phase (OD_600_ ≈ 1.0); cells were harvested by centrifugation (5000× *g*, 10 min, 4 °C), washed twice and resuspended in sterile 0.85% NaCl, and the suspension was adjusted to 1 × 10^11^ CFU mL^−1^ using an OD–CFU calibration curve and verified by plate counting. For the fungal inoculum, *Fusarium solani* (Fs) was cultured on PDA plates at 25 °C in the dark for 7 days to obtain abundant conidia; plates were flooded with sterile distilled water containing 0.05% (*v*/*v*) Tween 20, gently scraped, and the resulting suspension was filtered through sterile gauze, adjusted to 1 × 10^7^ spores mL^−1^ based on hemocytometer counts, and checked for spore viability (>95%) by germination tests and CFU counts on PDA. The CK treatment received 100 mL of sterile water applied as a soil drench to the root zone by slowly pouring the solution onto the soil surface around the stem base and distributing it evenly to promote uniform infiltration into the upper soil profile. The dxk33 treatment received 100 mL of the *B. mojavensis* dxk33 suspension (1 × 10^11^ CFU mL^−1^; ~1 × 10^13^ CFU per pot, 14.3 mL kg^−1^ soil), and the Fs treatment received 100 mL of the *F. solani* spore suspension (1 × 10^7^ spores mL^−1^; ~1 × 10^9^ spores per pot), both applied by soil drenching in the same manner. In the combined treatment (dxk33 + Fs), seedlings were first drenched with 100 mL of the dxk33 suspension and then inoculated 3 days later with 100 mL of the Fs spore suspension, using the same procedure to ensure a comparable distribution of inoculum in the root zone. This 3-day interval was chosen as a short preventive pre-inoculation period to allow initial establishment of dxk33 in the rhizosphere and the onset of early plant responses, while keeping the temporal offset between treatments small and consistent with practical application scenarios; bacterial colonization dynamics were not directly monitored prior to pathogen challenge, and this regime should therefore be interpreted as an early-stage preventive application rather than full equilibrium establishment of the biocontrol strain. Throughout the experimental period, soil moisture was maintained by daily weighing of the pots and replenishment with deionized water, and seedling positions in the growth chamber were randomized daily to minimize positional bias; the chamber was supplied with filtered fresh air distributed evenly through four corner inlets to ensure uniform environmental conditions. After the 30-day inoculation period, disease progression was systematically evaluated and plant growth parameters were measured to assess treatment effects on seedling growth performance and disease response.

### 2.3. Measurement of Plant Growth Traits, Disease Assessment, and Sample Collection

At 30 days after inoculation, plant growth parameters (height, fresh weight and dry weight) of each *C. lanceolata* seedling were recorded, and the incidence and severity of root rot were evaluated. Disease severity was scored on a 0–4 ordinal scale using two components, foliar symptoms (percentage of leaf area affected) and root necrosis (percentage of roots necrotic). Each component was first assigned to a category using the same thresholds: 1 (foliar ≤10%/root ≤25%), 2 (foliar 11–30%/root 26–50%), 3 (foliar 31–60%/root 51–75%), and 4 (foliar >60%/root >75% or plant death). The final disease severity score for a seedling was defined as the higher (more severe) of the two component categories, to avoid underestimating disease when symptoms were discordant between above- and below-ground tissues. For example, 15% foliar symptoms (category 2) combined with 60% root necrosis (category 3) was scored as 3. A disease index (DI, %) was then calculated as the mean severity score (0–4) relative to the maximum score (4) and expressed as a percentage. Disease severity was classified into five distinct levels based on symptom progression, ranging from level 0 (plants growing normally with green leaves and no visible symptoms) to level 4 (plants completely dead with no vitality). Disease severity scores of 1–3 represented progressively more pronounced symptoms, from slight leaf lesions and chlorosis to extensive yellowing, wilting and growth suppression, as defined by the quantitative severity scale described in the Methods. The disease index (DI) was subsequently calculated from this severity scale to quantify overall disease impact across treatments. Furthermore, the control efficacy (CE) of the biocontrol treatments was determined by comparing the disease index of treated groups to that of the control group. Following the completion of agronomic and disease assessments, seedlings were carefully uprooted. Loosely adhering bulk soil was removed by gently shaking the roots 2–3 times, and the soil that remained attached to the root surface was operationally defined as rhizosphere soil. This adhering soil was then dislodged by more vigorous shaking and gentle brushing into sterile 50 mL tubes and stored at −80 °C for subsequent DNA extraction and high-throughput sequencing of microbial communities. Concurrently, bulk soil samples were randomly collected from each treatment, thoroughly mixed, and air-dried for subsequent analysis of soil physicochemical properties and enzyme activities.

The disease index (DI, %) was calculated using the following formula:

$$\mathrm{DI}\;(\%)\;=\;\Sigma (S_{i}\times N_{i})/(Smax\times N)\;\times \;100$$

where S_i_ is the disease severity rating for category i (0–4), N_i_ is the number of plants in category i, Smax is the maximum rating (4 in this study), and N is the total number of plants assessed.

Control efficacy (CE, %) of each treatment relative to the pathogen-only control was calculated as:

CE (%) = (DI_CK−DI_T)/DI_CK × 100

where DI_CK is the disease index of the pathogen-only control (Fs) and DI_T is the disease index of the treatment being evaluated.

### 2.4. Soil Physicochemical Properties and Enzyme Activities

To comprehensively assess soil fertility and the functional status of the rhizosphere, major soil physicochemical properties and key enzyme activities were determined following standard soil analysis procedures. Ammonium nitrogen (NH_4_^+^-N) and nitrate nitrogen (NO_3_^−^-N) were extracted with KCl and quantified using a continuous-flow analyzer [27,28]. Soil organic matter was measured by the potassium dichromate oxidation method [29]. Alkali-hydrolyzable (available) nitrogen was determined by the alkaline hydrolysis diffusion method [30]. Available phosphorus and available potassium were analysed by the molybdenum–antimony colorimetric method and flame photometry, respectively [31,32]. In parallel, soil enzyme activities were evaluated as sensitive indicators of nutrient cycling and microbial metabolic function. Catalase (CAT) activity was assayed by potassium permanganate titration based on the decomposition of H_2_O_2_, urease (URE) activity by the sodium phenol–sodium hypochlorite colorimetric method via quantification of released NH_4_^+^-N, invertase (INV) activity by the 3,5-dinitrosalicylic acid method via determination of reducing sugars produced from sucrose, and acid phosphatase (ACP) activity by the disodium phenyl phosphate colorimetric method [31,33,34]. Together, these physicochemical and enzymatic measurements provide an integrated view of soil nutrient dynamics and rhizosphere functional status in relation to root rot disease development.

### 2.5. DNA Extraction and Illumina MiSeq Sequencing of Rhizosphere Microbial Communities

Total genomic DNA was extracted from approximately 5 g of fresh rhizosphere soil using the E.Z.N.A.^®^ Soil DNA Isolation Kit (Omega Bio-tek, Inc., Norcross, GA, USA) according to the manufacturer’s protocol. Briefly, soil samples were suspended in potassium phosphate buffer at a sample-to-buffer ratio of 1:5 (*w*/*v*), followed by water bath sonication at 40 kHz for 10 min and shaking at 200 rpm for 30 min at 25 °C to ensure thorough detachment of microbial cells from soil particles. The resulting supernatant was centrifuged at 12,000× *g* for 10 min at 4 °C, and the pellet was resuspended in 1.0 mL of preheated CTAB extraction buffer and homogenized before proceeding with the DNA extraction [35]. DNA concentration and purity were determined using a NanoDrop ND-1000 UV-Vis spectrophotometer (Thermo Fisher Scientific, Waltham, MA, USA), and high-throughput sequencing of both bacterial and fungal communities was conducted on the Illumina MiSeq platform (San Diego, CA, USA) [36]. The fungal ITS1 region was amplified using primers ITS1F and ITS2R, while the bacterial 16S rRNA gene was amplified using primers 799F and 1193R, enabling comprehensive profiling of both microbial domains.

### 2.6. Quality Control and Community Analysis of High-Throughput Sequencing Data

Raw sequence data were first processed using QIIME (Quantitative Insights into Microbial Ecology, version 1.7.0; QIIME Development Team, University of Colorado at Boulder, Boulder, CO, USA) to remove low-quality reads. After quality control, denoising, sequence merging, and chimera removal, a total of 1,765,722 high-quality fungal sequences and 1,850,283 high-quality bacterial sequences were obtained, with average read lengths of 265 bp and 417 bp, respectively. Amplicon sequence variants (ASVs) were clustered using UPARSE (version 7.1) at a 100% sequence similarity threshold. Representative fungal ASV sequences were taxonomically assigned using the UNITE database (version 18.11), whereas bacterial ASVs were classified with the USEARCH consensus classifier (version 8.0) against the SILVA 16S rRNA reference database (version 132). Sequencing data analysis of the *C. lanceolata* rhizosphere microbial communities was performed by Shanghai OE Biotech Co., Ltd. (Shanghai, China). Finally, the sequencing data of fungal and bacterial communities have been deposited in the Genome Sequence Archive (GSA) of the National Genomics Data Center under the accession number CRA032912 and CRA032911.

### 2.7. Evaluation of Rhizosphere Microbial Antagonism Toward the Root Rot Pathogen F. solani

To evaluate the ability of rhizosphere microbial communities to directly suppress the growth of the root-rot pathogen *F. solani* (Fs), in vitro community-level antagonism assays were performed. Rhizosphere microbial communities were extracted from soil samples of each treatment using the soil-suspension procedure described above; briefly, 10 g of fresh rhizosphere soil were added to 90 mL of sterile 0.85% NaCl (1:10, *w*/*v*), shaken at 180 rpm for 30 min at 25 °C, allowed to settle for 5 min, and the supernatant was passed through two layers of sterile gauze to obtain a cell-rich microbial suspension. An aliquot of this suspension was then diluted 1:10 (*v*/*v*) in potato dextrose broth (PDB) to generate a working community inoculum. An Fs spore suspension was added to each community inoculum to achieve a final pathogen concentration of 1 × 10^7^ spores mL^−1^, a level commonly used in in vitro antagonism assays and applied uniformly across treatments to permit comparison of suppression effects. Aliquots of 100 μL of the resulting mixtures were spread onto PDA plates and incubated in the dark at 25 °C for 5 days. After incubation, the diameter of *F. solani* colonies was measured along two perpendicular axes and averaged for each plate. Six independent biological replicates were included per treatment to ensure statistical reliability. These assays provided a community-level functional readout of antagonistic potential, complementing our sequencing-based characterization of rhizosphere microbiomes.

### 2.8. Statistical Analyses

Statistical analyses were performed to compare differences among treatments and to relate environmental variables to microbial community structure. Statistical analyses were performed to compare differences among treatments and to relate environmental variables to microbial community structure. All univariate response variables (plant height, fresh and dry biomass, disease index and control efficacy, soil physicochemical properties, soil enzyme activities, and Shannon diversity) were continuous and were therefore analysed using generalized linear models with a Gaussian error distribution and identity link (i.e., equivalent to linear models), with treatment as a fixed factor, followed by Wald pairwise comparisons for post hoc tests (*p* < 0.05). Model adequacy was evaluated by inspecting residual-versus-fitted plots and normal Q–Q plots, and by testing residual normality (Kolmogorov–Smirnov test) and homoscedasticity (Levene’s test). When assumptions were violated, variables were log10-transformed and models were refitted. Because Poisson/binomial-type count models were not used for these univariate endpoints, overdispersion diagnostics were not applicable in this context. β-diversity patterns were visualized by principal coordinate analysis (PCoA) based on Bray–Curtis distance matrices, and treatment effects on community composition were evaluated using one-way analysis of similarity (ANOSIM). Redundancy analysis (RDA) was performed to examine the relationships between environmental variables and microbial community composition. Spearman’s rank correlation coefficients (ρ) were calculated to generate correlation heatmaps between environmental variables and microbial genera. Linear discriminant analysis effect size (LEfSe) was implemented using the Galaxy platform, applying a non-parametric Kruskal–Wallis test among treatments and Wilcoxon rank-sum tests between subclasses, with an LDA score threshold of 3.0 and *p* < 0.05. Functional profiles predicted by FUNGuild and PICRUSt2 were interpreted descriptively without additional hypothesis testing.

## 3. Results

### 3.1. Biocontrol Effect and Growth Promotion of B. mojavensis dxk33 Against Root Rot in C. lanceolata

Greenhouse pot experiments showed that inoculation with *Bacillus mojavensis* dxk33 markedly suppressed root rot in *Cunninghamia lanceolata* seedlings and improved overall growth performance. After 30 days, dxk33-inoculated seedlings exhibited vigorous growth and well-developed root systems. Quantitatively, plant height, fresh biomass, and dry biomass were 26.93%, 24.13%, and 41.03% higher than those of the uninoculated control, respectively (GLM followed by Wald pairwise comparisons, *p* < 0.05; mean ± SD in Table 1), indicating a pronounced growth-promoting effect under the present experimental conditions. In contrast, seedlings in the *Fusarium solani* (Fs) treatment displayed extensive leaf chlorosis and defoliation, severe root tip necrosis, and a drastic loss of lateral and fibrous roots; accordingly, all growth parameters were significantly reduced relative to the control, and the disease index reached 79.38 ± 0.83, consistent with severe infection and substantial physiological damage (Figure 1; Table 1). Notably, preventive application of dxk33 in the combined dxk33 + Fs treatment substantially mitigated disease and improved plant vigor: fresh and dry biomass were significantly higher than in the Fs-only group (*p* < 0.05), while the disease index decreased to 24.66 ± 1.12, corresponding to a control efficacy of 68.93%. Detailed measurements of plant growth and disease parameters under each treatment are summarized in Table 1.

### 3.2. Effects of B. mojavensis dxk33 on Soil Nutrients and Enzyme Activities in the Rhizosphere of C. lanceolata

Inoculation with *B. mojavensis* dxk33 markedly enhanced both soil nutrient status and enzymatic activity in the rhizosphere of *C. lanceolata*. Compared with the control, the dxk33 treatment significantly increased ammonium nitrogen, nitrate nitrogen, soil organic matter, available nitrogen, available phosphorus and available potassium by 60.72, 40.48, 45.95, 29.40, 51.90 and 20.70%, respectively (*p* < 0.05). By contrast, *F. solani* (Fs) inoculation induced a contrasting pattern in soil nitrogen fractions and other nutrients: AMN and NN increased by 42.40% and 34.12%, respectively, whereas SOM, AN, AP and AK decreased by 35.56, 39.15, 21.61 and 30.60%. This combination of higher inorganic N but depleted organic matter and alkali-hydrolysable N suggests that pathogen infection accelerates N mineralization and short-term accumulation of inorganic N, while simultaneously exhausting more stable nutrient pools. Notably, under the combined dxk33 + Fs treatment, all six nutrient parameters were significantly higher than in the Fs-only group, with respective increases of 7.28, 14.85, 99.35, 53.94, 48.79 and 40.49% (*p* < 0.05), indicating that dxk33 effectively mitigated pathogen-induced nutrient depletion. For soil enzyme activities, dxk33 inoculation enhanced catalase, urease, invertase and acid phosphatase by 13.17, 19.60, 31.35 and 31.03%, respectively, compared with the control (*p* < 0.05), consistent with a stimulation of rhizosphere biochemical functioning. In contrast, Fs infection significantly reduced these enzyme activities by 20.00, 12.94, 23.89 and 37.59%, indicating an overall impairment of key processes involved in C, N and P cycling under root-rot stress, likely related to root necrosis, reduced rhizodeposition and the loss of beneficial microbial groups. Co-inoculation with dxk33 and Fs substantially restored enzyme activities, with increases of 18.26, 15.59, 48.85 and 88.90%, respectively, relative to Fs alone. The effects of the different treatments on rhizosphere nutrient pools and soil enzyme activities are summarized in Figure 2 and Figure 3.

Collectively, these results indicate that *B. mojavensis* dxk33 not only counteracts pathogen-induced nutrient depletion but also mitigates the inhibition of key soil enzymes, thereby contributing to a more favorable soil microenvironment that supports the healthy growth and resilience of *C. lanceolata* (Figure 2 and Figure 3). Taken together, the nutrient and enzyme data demonstrate that soil physicochemical properties and biochemical functioning differed significantly among treatments, providing an environmental context for the treatment-specific rhizosphere microbial communities described below.

### 3.3. In Vitro Antagonistic Activity of Bacillus Mojavensis dxk33 Against Fusarium Solani

**Figure 4:**
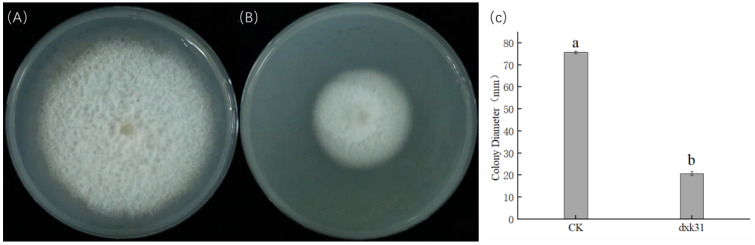
Antagonistic effect of *Bacillus mojavensis* dxk33 on the mycelial growth of the root-rot pathogen *Fusarium solani* (Fs) associated with *Cunninghamia lanceolata*. (**A**) Fs cultured alone, showing complete colony expansion with dense hyphae covering the entire plate after 7 days at 25 °C in the dark. (**B**) Fs co-cultured with *B. mojavensis* dxk33, showing strongly inhibited colony expansion, with a markedly reduced colony diameter and sparse hyphae at the colony margin. (**C**) Quantitative comparison of colony diameter between treatments. Different letters above the bars denote significant differences between treatments (one-way ANOVA followed by Tukey’s HSD test, *p* < 0.05), and values are presented as mean ± standard error.

The in vitro antagonism assay revealed that the growth of *Fusarium solani* (Fs), the causal agent of root rot in *C. lanceolata*, was markedly suppressed in culture medium supplemented with *Bacillus mojavensis* dxk33 suspension. In the control treatment, Fs colonies reached a diameter of 75.77 ± 0.55 mm, whereas the addition of dxk33 reduced the colony diameter to 20.70 ± 0.83 mm, representing a 72.68% inhibition rate. Furthermore, the hyphal expansion of Fs on dxk33-amended plates was visibly slower and sparser than that in the control, with significantly reduced mycelial density and structural integrity at the colony margins. Collectively, these results demonstrate that *Bacillus mojavensis* dxk33 has pronounced antifungal activity against *Fusarium solani* under in vitro conditions. This dual-culture assay therefore provides direct functional evidence that dxk33 can inhibit the pathogen in the absence of the plant host and a surrounding microbial community, supporting the view that direct antagonism is an important component of its biocontrol activity in *Cunninghamia lanceolata*, even though the present study does not quantify its relative contribution in planta (Figure 4).

### 3.4. Diversity and Composition of Rhizosphere Microbial Communities in C. lanceolata

**Figure 5:**
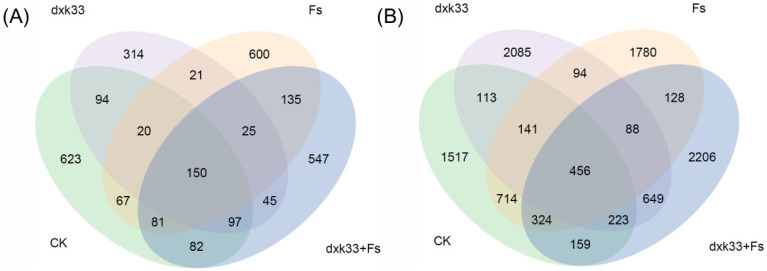
Venn diagrams illustrating the number of unique and shared amplicon sequence variants (ASVs) of fungal and bacterial communities in the rhizosphere of *C. lanceolata* under different treatments. (**A**) Rhizosphere fungal communities; (**B**) Rhizosphere bacterial communities. CK: uninoculated control; Fs: inoculation with *F. solani*; dxk33: inoculation with *B. mojavensis* dxk33; dxk33 + Fs: co-inoculation with *B. mojavensis* dxk33 and *F. solani*.

High-throughput sequencing of the rhizosphere microbial communities of *C. lanceolata* under different treatment conditions yielded 2933 fungal ASVs and 10,799 bacterial ASVs, revealing distinct responses of these microbial domains to the experimental treatments. Taxonomic classification demonstrated substantially greater phylogenetic diversity in bacteria, with fungal ASVs distributed across 13 phyla, 38 classes, 94 orders, 174 families, 309 genera, and 407 species, while bacterial ASVs spanned 35 phyla, 99 classes, 249 orders, 404 families, 714 genera, and 1190 species. Venn diagram analysis further revealed fundamental differences in community assembly patterns, with fungal communities showing pronounced compositional variation across treatments, as evidenced by only 150 shared ASVs, whereas bacterial communities exhibited greater compositional overlap, with 456 ASVs shared across all treatments (Figure 5).

### 3.5. Alpha Diversity and Compositional Variation in Rhizosphere Microbial Communities in Cunninghamia lanceolata

**Figure 6:**
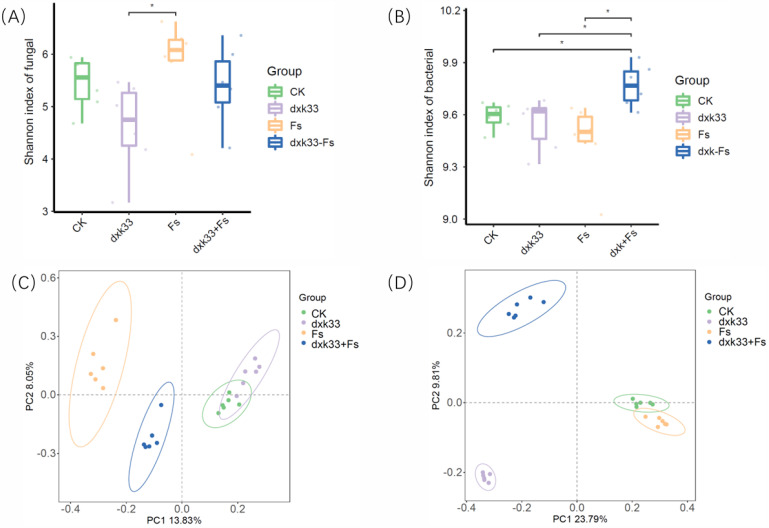
Alpha-diversity and community structural differences of rhizosphere microbial communities in *C. lanceolata*. (**A**,**B**) Shannon diversity indices of rhizosphere fungal (**A**) and bacterial (**B**) communities under different treatments. (**C**,**D**) Principal coordinate analysis (PCoA) based on Bray–Curtis distances, illustrating compositional differences in fungal (**C**) and bacterial (**D**) communities. CK: uninoculated control; Fs: inoculated with the pathogenic fungus *F. solani*; dxk33: inoculated with the antagonistic bacterium *B. mojavensis* dxk33; dxk33 + Fs: co-inoculation with the antagonistic bacterium and *F. solani*. *: Indicates statistically significant differences (*p* < 0.05).

Analysis of the Shannon index, a key indicator of α-diversity that integrates both species richness and evenness, revealed distinct treatment effects on the structural complexity of rhizosphere microbial communities. The control group exhibited the highest Shannon indices for both fungal and bacterial communities, indicating greater microbial diversity in healthy soils, whereas infection by *F. solani* (Fs) significantly reduced both fungal and bacterial diversity, demonstrating pathogen-induced disruption of the rhizosphere microecosystem. Interestingly, inoculation with *B. mojavensis* dxk33 alone led to a marked reduction in fungal community diversity, suggesting competitive inhibition of certain fungal taxa, but under pathogen stress, co-inoculation with dxk33 and Fs partially restored fungal diversity while significantly enhancing bacterial diversity. Notably, the dxk33 + Fs group exhibited the highest bacterial Shannon index among all treatments, significantly exceeding those of the CK, dxk33, and Fs groups (*p* < 0.05), indicating that ecological interactions between the antagonist and pathogen improved bacterial richness and evenness. Principal coordinate analysis (PCoA) based on Bray–Curtis distances further illustrated differences in β-diversity, with distinct separation among treatments indicating substantial reshaping of microbial community composition. Fungal communities showed greatest deviation in the Fs treatment, while the dxk33 + Fs group occupied an intermediate position, suggesting partial recovery of community composition toward the healthy control state (Figure 6).

### 3.6. Modulatory Effects of B. mojavensis dxk33 on Rhizosphere Microbial Community Structure of C. lanceolata Under F. solani Infection

**Figure 7:**
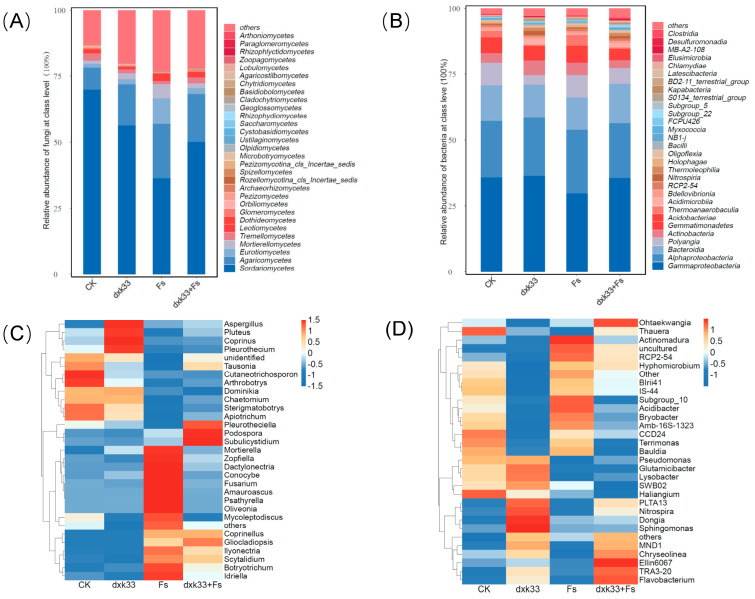
Relative abundance and hierarchical clustering of fungal and bacterial communities in the rhizosphere of *C. lanceolata* under different treatments. (**A**) Relative abundance of fungal communities at the class level. (**B**) Relative abundance of bacterial communities at the class level. Taxa with relative abundances below 0.5% of the total sequence count were grouped as “Others”. (**C**) Heatmap and hierarchical clustering of fungal communities at the genus level, displaying the 30 most abundant genera. (**D**) Heatmap and hierarchical clustering of bacterial communities at the genus level, displaying the 30 most abundant genera. Clustering analyses were performed based on Bray–Curtis distances using the average linkage method. CK: uninoculated control; Fs: inoculated with *F. solani*; dxk33: inoculated with *B. mojavensis* dxk33; dxk33 + Fs: co-inoculation with *B. mojavensis* dxk33 and *F. solani*.

Analysis of microbial community composition at the class level revealed that both *B. mojavensis* dxk33 and *F. solani* (Fs) significantly altered the structure of rhizosphere microbial communities. In the fungal community, the dominant classes were *Sordariomycetes*, *Agaricomycetes*, *Eurotiomycetes* and *Mortierellomycetes*, which collectively accounted for the majority of total abundance. The control group was predominantly characterized by Sordariomycetes, whereas *F. solani* infection caused a 54.33% decrease in its relative abundance compared with the control (*p* < 0.05), accompanied by marked increases in *Agaricomycetes* and *Eurotiomycetes*. While dxk33 treatment alone also slightly reduced *Sordariomycetes* abundance, the combined dxk33 + Fs treatment substantially restored it, achieving a 49.49% increase compared with Fs treatment alone. In the bacterial community, dominated by *Gammaproteobacteria*, *Alphaproteobacteria*, *Bacteroidia*, and *Actinobacteria*, *F. solani* infection reduced *Gammaproteobacteria* by 16.99% while increasing *Actinobacteria* by 30.56%. dxk33 inoculation increased *Alphaproteobacteria* abundance by 3.79% compared with CK, with this enhancement being more pronounced in the dxk33 + Fs treatment, while effectively suppressing the proliferation of *Actinobacteria* and *Bacteroidia*. At the genus level, *F. solani* infection significantly enriched potential pathogens such as *Fusarium*, *Podospora*, *Pleurotheciella*, *Aspergillus*, and *Chaetomium*, while depleting beneficial genera including *Mortierella* and *Trichoderma*. In contrast, dxk33 inoculation enriched *Chaetomium*, *Mortierella*, and the nematophagous fungus *Arthrobotrys*. Under co-inoculation conditions, the relative abundance of *Fusarium* decreased while beneficial genera such as *Mortierella*, *Chaetomium*, and *Aspergillus* were partially restored. Similarly, Fs treatment induced significant structural shifts in the bacterial community, increasing the relative abundance of *Flavobacterium*, *Chryseolinea*, *Nitrospira*, and *Acidibacter* while reducing plant growth-promoting genera such as *Pseudomonas*, *Sphingomonas*, and *Lysobacter*. dxk33 inoculation enriched these beneficial bacterial taxa, and in the dxk33 + Fs treatment, the relative abundance of *Pseudomonas* and *Sphingomonas* recovered significantly compared with the Fs manuscript (*p* < 0.05), while pathogen-associated genera declined (Figure 7).

### 3.7. Associations Between Rhizosphere Soil Physicochemical Properties, Enzyme Activities, and Microbial Community Structure

**Figure 8:**
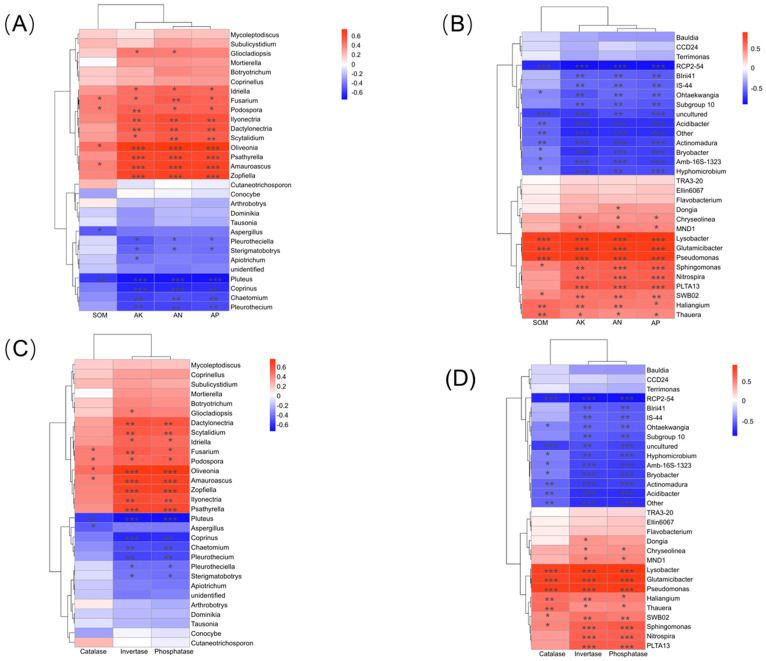
Spearman correlation heatmaps illustrating the relationships between environmental factors and the rhizosphere microbial communities of *C. lanceolata*. (**A**) Correlations between soil physicochemical properties and rhizosphere fungal communities at the genus level. (**B**) Correlations between soil physicochemical properties and rhizosphere bacterial communities at the genus level. (**C**) Correlations between soil enzyme activities and rhizosphere fungal communities at the genus level. (**D**) Correlations between soil enzyme activities and rhizosphere bacterial communities at the genus level. Experimental treatments included four groups: CK (uninoculated control), Fs (*F. solani* inoculation), dxk33 (*B. mojavensis* dxk33 inoculation), and dxk33 + Fs (co-inoculation). Asterisks indicate significance levels: * 0.01 < *p* ≤ 0.05; ** 0.001 < *p* ≤ 0.01; *** *p* ≤ 0.001.

Both fungal and bacterial communities in the rhizosphere of *C. lanceolata* exhibited significant correlations with soil physicochemical properties and enzyme activities. For the fungal community, available nitrogen, available phosphorus, and available potassium showed strong positive correlations with the relative abundances of most fungal genera. Genera such as *Fusarium*, *Mortierella*, *Podospora*, and *Ilyonectria* were significantly enriched under nutrient-rich conditions (*p* < 0.05). In contrast, pH showed significant negative correlations with *Chaetomium*, *Aspergillus*, *Pluteus*, and *Pleurothecium* (*p* < 0.05). Regarding enzyme activities, catalase, invertase, and phosphatase exhibited strong positive correlations (*p* < 0.05–0.001) with the abundances of several fungal genera, including *Mortierella*, *Fusarium*, *Ilyonectria*, *Podospora*, *Gliocladiopsis*, and *Psathyrella*. AN, AP, and AK showed positive correlations (*p* < 0.05–0.001) with classical plant growth-promoting and antagonistic genera, including *Pseudomonas*, *Sphingomonas*, *Lysobacter*, *Glutamicibacter*, *Nitrospira*, *Thauera*, and *Haliangium*, all of which also showed positive associations with soil organic matter, indicating that nutrient- and carbon-rich environments favor the enrichment of beneficial bacteria. Conversely, oligotrophic or acidophilic genera such as RCP2-54, *Actinomadura*, *Hyphomicrobium*, *Acidibacter*, *Bryobacter*, and *Terrimonas* were negatively correlated with AN, AP, and AK but positively correlated with lower pH values. At the enzymatic level, catalase, invertase, and phosphatase activities were strongly and positively correlated with the abundances of beneficial bacterial taxa (*p* < 0.01–0.001) (Figure 8).

### 3.8. Effect of Root Rot Disease on the Rhizosphere Microbial Taxonomy of C. lanceolata

**Figure 9:**
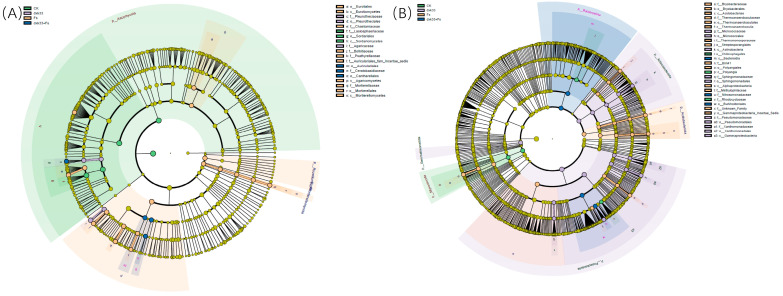
Differential taxa in rhizosphere microbial communities of *C. lanceolata* under different treatments. Cladograms based on linear discriminant analysis effect size (LEfSe) illustrate significantly enriched taxa at the class, order, family, and genus levels among the CK, Fs, dxk33, and dxk33 + Fs treatments. (**A**) Fungal communities; (**B**) Bacterial communities. Taxa with a logarithmic LDA score > 3 and *p* < 0.05 were considered significantly discriminative.

Linear discriminant analysis coupled with effect size was employed to identify taxonomic differences in the rhizosphere microbial communities of *C. lanceolata* under the four treatments, revealing distinct phylogenetic and functional shifts among groups. In the fungal community, pronounced phylogenetic differentiation was observed across treatments, with the control group enriched with *Sordariomycetes*, *Sordariales*, and *Lasiosphaeriaceae*. In contrast, *Fusarium solani* infection led to substantial enrichment of *Eurotiomycetes*, *Agaricomycetes*, and *Mortierellomycetes*, along with orders *Eurotiales* and *Mortierellales* and families such as *Bolbitiaceae*, *Psathyrellaceae*, *Auriculariales*, and *Mortierellacea*. Inoculation with *B. mojavensis* dxk33 significantly enriched *Pleurotheciales*, *Pleurotheciaceae*, and *Agaricaceae* (*p* < 0.05). In the co-inoculation treatment, basidiomycetous taxa including *Auriculariales*, *Cantharellales*, and *Ceratobasidiaceae* were dominant and exhibited the highest LDA scores. In bacterial communities, LEfSe analysis revealed equally clear treatment-specific patterns, with the control group enriched in *Polyangia*, *Chitinophagales*, and *Rhodocyclaceae*, representing predator- and decomposer-type bacteria typical of healthy rhizospheres. Fs treatment enriched *Acidobacteriae*, *Thermoanaerobaculia*, and *Alphaproteobacteria* classes, along with orders *Bryobacterales*, *Thermoanaerobaculales*, *Streptosporangiales*, *Polyangiales*, and *Gammaproteobacteria*, with families including *Bryobacteraceae*, *Thermoanaerobaculaceae*, *Blrii41*, and *Methylophilaceae* significant increased (*p* < 0.05). dxk33 inoculation significant enriched *Actinobacteria* and *Gammaproteobacteria* and their subordinate orders *Micrococcales*, *Sphingomonadales*, *Pseudomonadales*, and *Xanthomonadales*, with corresponding increases in *Micrococcaceae*, *Sphingomonadaceae*, *Pseudomonadaceae*, and *Xanthomonadaceae* (*p* < 0.05). Under co-inoculation conditions, enrichment of *Bacteroidia*, *Burkholderiales*, and *Nitrosomonadaceae* (Figure 9).

### 3.9. Functional Prediction of Rhizosphere Fungal and Bacterial Communities Under Different Treatments

**Figure 10:**
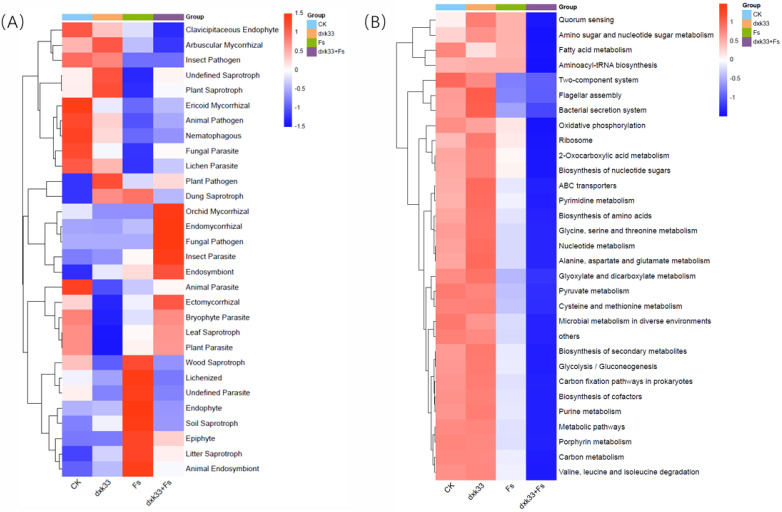
Functional prediction of rhizosphere fungal and bacterial communities of *C. lanceolata* under different treatments (CK, Fs, dxk33, and dxk33 + Fs). (**A**) Functional composition of rhizosphere fungal communities predicted by FUNGuild, illustrating the ecological roles and compositional differences in fungal communities among treatments. (**B**) Heatmap showing the top 30 most abundant KEGG pathways in rhizosphere bacterial communities predicted by PICRUSt2, identifying major metabolic pathways differentially represented across treatments.

FUNGuild-based functional annotation revealed that different treatments profoundly reshaped the functional composition of rhizosphere fungal communities associated with *C. lanceolata*. In the control group, the community was apparently dominated by saprotrophic taxa including plant, soil, wood, and litter saprotrophs, accompanied by moderate proportions of endophytic and mycorrhizal fungi. Upon addition of *F. solani*, the predicted functional structure shifted dramatically, with pathogenic and parasitic guilds significantly enriched (*p* < 0.05), while saprotrophic and mycorrhizal groups declined sharply. In contrast, inoculation with *B. mojavensis* dxk33 under pathogen-free conditions may enhance the relative abundance of mycorrhizal and endophytic fungi while suppressing pathogenic and parasitic taxa. In the co-inoculation treatment, the fungal community exhibited pathogenic/parasitic guilds markedly. Based on PICRUSt2 predictions, bacterial functional profiles similarly varied significantly among treatments (*p* < 0.05), with CK and dxk33 treatments predicting high metabolic activity characterized by significant enrichment of core biosynthetic and energy pathways including carbon metabolism, glycolysis/gluconeogenesis, fatty acid metabolism, amino acid and nucleotide biosynthesis, and purine/pyrimidine metabolism (Figure 10).

## 4. Discussion

Although our study provides a mechanistic picture of how *B. mojavensis* dxk33 suppresses *F. solani*–induced root rot and reshapes the rhizosphere microbiome, we emphasize that all experiments were performed on three-year-old *C. lanceolata* seedlings grown in a single soil type under controlled greenhouse and growth-chamber conditions. Thus, our results should be viewed as system-specific proof-of-concept rather than universally transferable prescriptions. The extent to which dxk33 or related *Bacillus* strains will reproduce similar disease-suppressive effects in other tree species, crops, or soils with contrasting texture, pH, organic matter content, and background microbiota remains unknown [37]. Host genotype and soil properties are known to strongly modulate inoculant establishment, rhizosphere community assembly, and biocontrol efficacy [38]. Future work should therefore include multi-site and multi-host experiments, particularly field trials across different soil types and *C. lanceolata* provenances or other forest tree species, to evaluate the robustness and scope of dxk33-based microbiome engineering strategies.

Under our greenhouse microcosm conditions, inoculation with *B. mojavensis* dxk33 was associated with reduced severity of *F. solani*–related root rot in *C. lanceolata*, together with changes in plant performance, soil properties, and the rhizosphere microbiome. Taken together, these observations are consistent with the idea that dxk33 can contribute to disease suppression in this system through a combination of direct and indirect effects [26,39]. However, our data do not allow us to disentangle the relative contributions of potential mechanisms such as direct antagonism, resource competition, modulation of plant physiology, or microbiome-mediated interactions, and we cannot claim a single, fully resolved ecological strategy. Instead, we interpret our results as evidence that dxk33 acts as one influential component within the rhizosphere, whose presence coincides with shifts toward plant and community states that are typically associated with disease-suppressive conditions [40].

In our experiments, dxk33-treated seedlings showed both higher biomass and lower disease scores compared with the pathogen-only treatment, and in some cases also compared with the uninoculated control. Under the conditions tested here, dxk33 inoculation was associated with higher soil nutrient availability and enhanced activities of key C-, N-, and *p*-cycling enzymes, and these patterns coincided with improved plant growth. These results suggest that changes in rhizosphere nutrient status may form part of the growth-promoting effect of dxk33, although we did not directly measure host physiological responses such as hormone levels, root exudation, or defense gene expression. Likewise, the mitigation of disease symptoms in the co-inoculation treatment is compatible with the hypothesis that dxk33 may prime or support plant defense responses and thus contribute to a form of induced resistance, but this remains to be demonstrated experimentally [41]. Our findings therefore align with established concepts that plant-beneficial rhizobacteria can simultaneously influence growth and defense, while highlighting that the specific molecular and physiological mechanisms underlying the dxk33-*C. lanceolata* interaction are still unresolved.

At the soil level, dxk33 inoculation coincided with higher levels of soil organic matter, available nitrogen, phosphorus, and potassium, and with increased activities of catalase, urease, invertase, and acid phosphatase relative to the pathogen-only treatment. These patterns indicate that dxk33 is associated with a more active rhizosphere biochemical environment, which may be less favorable for pathogen proliferation and more supportive of beneficial microorganisms. Previous work has shown that beneficial rhizobacteria can enhance soil enzyme activities and nutrient availability, and that such changes can contribute to the emergence of disease-suppressive soils [42,43]. In our study, the concurrent increases in nutrient availability and enzyme activities, together with reduced root-rot severity, are consistent with this framework and suggest that improved nutrient cycling may be one contributing factor in dxk33-mediated disease suppression. Nevertheless, these associations are correlative, and additional experiments will be required to determine the causal links between enzyme activity, nutrient status, microbial composition, and disease outcomes.

Amplicon sequencing further indicated that dxk33 inoculation coincided with pronounced shifts in both fungal and bacterial community composition, particularly under pathogen stress. In the presence of *F. solani*, dxk33-treated rhizosphere communities differed from pathogen-only communities and, in several respects, more closely resembled the communities found in healthy controls. We interpret these patterns as evidence that dxk33 influences the trajectory of community assembly under pathogen pressure, but we cannot resolve from these data whether dxk33 actively “engineers” the microbiome, passively benefits from pre-existing structures, or both. The enrichment of bacterial taxa that are frequently reported as plant growth-promoting or antagonistic (e.g., *Pseudomonas*, *Sphingomonas*, *Lysobacter*), together with reduced relative abundance of certain pathogenic or potentially pathogenic fungal genera (including *Fusarium*), is consistent with a shift towards a more protective microbial consortium. However, we recognize that our approach captures relative abundances in bulk rhizosphere samples and does not directly quantify functional interactions or spatial organization at the root–soil interface. We further note that amplicon sequencing data are compositional: changes in relative abundance do not necessarily reflect changes in absolute population size, because a taxon can decline in relative abundance while increasing in absolute abundance if total community size increases. Accordingly, throughout the manuscript we interpret sequencing results as shifts in community composition (relative abundance) rather than absolute microbial loads. Quantifying absolute abundances (e.g., via qPCR of total bacteria/fungi and key taxa, or spike-in standards) would be a valuable next step to corroborate the population-level dynamics suggested by our compositional patterns.

The functional interpretations presented here are based on predicted functional profiles rather than direct measurements. Fungal functional guilds were inferred using FUNGuild [44], and bacterial functional pathways were predicted using PICRUSt2 [45,46]. These tools provide useful hypotheses about the potential ecological roles of taxa, but they necessarily rely on existing databases and assumptions, and they should not be regarded as definitive evidence of actual in situ functions. In our data, the dxk33 + Fs treatment showed a predicted increase in saprotrophic and mycorrhizal fungal guilds and a corresponding decrease in pathogenic and parasitic guilds compared with the pathogen-only treatment, which we interpret as a possible functional rebalancing towards mutualistic and decomposer roles. Similarly, predicted bacterial functions indicated higher representation of core metabolic pathways, nutrient transport systems, and cell–cell communication (e.g., quorum sensing) under dxk33 inoculation compared with pathogen-only conditions [47,48]. These patterns are consistent with the idea that dxk33 may help to maintain or restore functional redundancy and connectivity in the rhizosphere microbiome [49]. Nevertheless, because these inferences are model-based, they should be treated as tentative and require confirmation through targeted metatranscriptomic, metaproteomic, or metabolomic analyses.

Correlation analyses between environmental variables and dominant taxa provided additional, albeit indirect, insight into the ecological context of dxk33-mediated effects. Associations between higher nutrient levels, elevated enzyme activities, and the increased relative abundance of several beneficial bacterial and fungal taxa are in line with a scenario in which dxk33 participates in a positive feedback loop linking plant performance, soil biochemistry, and microbiome composition. However, these analyses are inherently correlational and do not establish causal direction; alternative explanations, such as shared responses of plants and microbes to underlying soil conditions, remain possible. We therefore interpret these patterns as supporting, but not proving, the notion that dxk33 contributes to the emergence of a more functionally robust, disease-suppressive rhizosphere state.

From an applied perspective, our results suggest that *B. mojavensis* dxk33 has the potential to be developed as a component of integrated management strategies for soil-borne diseases in *C. lanceolata* plantations, particularly in monoculture systems where root rot can cause substantial economic and ecological losses. The combination of in vitro antagonism, improved plant performance, and characteristic microbiome shifts observed under our experimental conditions makes dxk33 a promising candidate for further evaluation. However, we emphasize that our study was conducted in controlled greenhouse microcosms with preventive co-inoculation of the pathogen and the biocontrol strain, and that field conditions are likely to be more complex. Long-term persistence, performance across soil types and climates, and potential interactions with indigenous microbial communities will need to be rigorously tested before practical recommendations can be made.

Our experimental design has several important limitations that should be considered when interpreting the results. First, *F. solani* and *B. mojavensis* dxk33 were introduced simultaneously or within a short time window, reflecting a preventive application scenario rather than a curative one. Under field conditions, pathogens may already be established and causing root damage before any biocontrol agent is applied, and the efficacy of dxk33 under such circumstances remains unknown. Second, our conclusions are based on short-term responses in a single soil type and host genotype, and it is unclear how generalizable these patterns are across different stands, soils, or climatic conditions [50]. Third, we did not directly quantify dxk33 populations in the rhizosphere over time, nor did we measure specific plant defense responses or microbial metabolites, which limits our ability to link particular processes to the observed outcomes. Finally, the functional interpretations derived from FUNGuild and PICRUSt2 are predictive and correlative and should be viewed as working hypotheses rather than established mechanisms.

Future studies should therefore focus on disentangling the relative contributions of direct pathogen inhibition, plant-mediated defense responses, and microbiome-mediated interactions in dxk33-induced disease suppression. Sequential inoculation experiments, in which *F. solani* is allowed to establish prior to dxk33 application, would help to assess the performance of dxk33 under more curative scenarios. Combining amplicon sequencing with metatranscriptomics, metabolomics, and targeted assays of plant defense pathways could provide more direct evidence for the mechanisms suggested by our predictive functional analyses. Field trials across multiple sites and management regimes will also be essential to determine the robustness and practicality of dxk33-based interventions in real forestry systems.

In conclusion, our work provides an ecological and microbiome-centered perspective on biological control in *C. lanceolata* plantations. Under the conditions tested, dxk33 inoculation was associated with reduced root-rot severity, improved plant performance, altered soil biochemical properties, and characteristic changes in rhizosphere microbial communities. These findings support the broader concept that effective disease suppression may involve coordinated changes across multiple components of the plant–soil–microbe system, rather than relying solely on direct pathogen inhibition. At the same time, we recognize that many of the mechanistic links proposed here remain hypothetical and rely on correlative and predictive analyses. Further experimental work will be required to validate these hypotheses and to translate dxk33-based microbiome interventions into robust, field-ready strategies for sustainable forest management.

## 5. Conclusions

In summary, *B. mojavensis* dxk33 suppresses *F. solani*-induced root rot in *C. lanceolata* through an integrated ecological strategy that combines direct antagonism with extensive restructuring of the rhizosphere microbiome. In our experiments, dxk33 treatment coincided with mitigation of pathogen-induced dysbiosis, restoration of soil nutrient cycling and enzymatic activities, and enhancement of plant physiological fitness and defense capacity, together supporting the reassembly of a more resilient and functionally robust microbial community that is associated with reduced disease. These multi-layered effects underscore dxk33′s dual role as both a growth-promoting and biocontrol agent, functioning as an ecological engineer that is associated with coordinated shifts in plant performance, soil biochemical properties, and rhizosphere microbiome structure that coincide with reduced disease. Given its strong microbiome-modulating capacity and compatibility with indigenous beneficial taxa, dxk33 represents a promising tool for sustainable management of soil-borne diseases in forest plantations. Future work should focus on field-scale validation, elucidation of molecular mechanisms underlying plant–microbe signaling, and optimization of application strategies to support practical deployment in forestry systems. We also note that the observed links between microbiome reconfiguration and disease suppression are correlative in this study and warrant future experiments explicitly testing causality.

## Figures and Tables

**Table 1:** Effect of *B. mojavensis* dxk33 on plant growth and root rot disease in *C. lanceolata* under pot culture conditions.

Treatment	Plant Height (cm)	Root Fresh Weight (kg)	Root Dry Weight (kg)	Disease Index	Control Efficacy (CE) (%)
ck	33.47 ± 0.82 b	1.24 ± 0.08 b	0.26 ± 0.05 b	0.00 ± 0.00 c	–
dxk33	42.49 ± 1.14 a	1.54 ± 0.07 a	0.37 ± 0.05 a	0.00 ± 0.00 c	–
Fs	22.06 ± 1.39 d	0.81 ± 0.05 c	0.2 ± 0.04 b	79.38 ± 0.83 a	0.00
dxk33 + Fs	28.44 ± 1.07 c	1.13 ± 0.14 b	0.27 ± 0.05 b	24.66 ± 1.12 b	68.93

Note: Values are means ± SD. Different lowercase letters within the same column indicate significant differences among treatments (one-way ANOVA followed by Tukey’s multiple comparison test, *p* < 0.05). CE, control efficacy.

## Data Availability

The raw sequencing data generated in this study have been deposited in the Genome Sequence Archive (GSA) under the accession numbers CRA032912 and CRA032911. All other data supporting the findings of this study are included in the article.
